# Comparison of neonatal outcomes and live-birth defects after progestin-primed ovarian stimulation versus conventional ovarian stimulation for in vitro fertilization: A large retrospective cohort study

**DOI:** 10.1097/MD.0000000000011906

**Published:** 2018-08-24

**Authors:** Ningling Wang, Jiaying Lin, Qianqian Zhu, Yong Fan, Yun Wang, Yonglun Fu, Yanping Kuang

**Affiliations:** Department of Assisted Reproduction, Shanghai Ninth People's Hospital, Shanghai Jiaotong University School of Medicine, Shanghai, People's Republic of China.

**Keywords:** birth defects, congenital malformations, live birth, progestin-primed ovarian stimulation

## Abstract

Progestin-primed ovarian stimulation (PPOS) is a new ovarian stimulation regimen for in vitro fertilization (IVF), with the advantages of an oral administration route and more control over preovulatory luteinizing hormone (LH) levels. Assessing the safety of this novel regimen is an important premise for its routine practice.

We conducted a large retrospective cohort study for infants born between August 2014 and April 2017 from IVF and embryo transfer cycles after either PPOS and the conventional gonadotropin-releasing hormone-agonist (GnRH-a) short protocol at our center. Around 1589 live-born infants were finally enrolled, corresponding to 1258 frozen-thawed (FET) cycles, which led to 855 live-born infants from PPOS (659 FET cycles) and 734 live-born infants from the short protocol (599 FET cycles).

Birth characteristics regarding gestational age, birth weight and length, infant sex, and early neonatal death were comparable between the 2 groups. The incidence of live-birth defects in the PPOS group (1.52%) was similar to that in the short protocol group (1.63%) and was not statistically significant. For birth defects, the risk significantly increased for multiple births, and the adjusted odds ratio was 3.14 (95% confidence interval [CI]: 1.25–7.88). No associations were found between congenital birth defects and maternal age, body mass index (BMI), the duration of infertility, method of insemination, infant sex, embryo stage at transfer, the number of embryos transferred or ovarian stimulation regimen.

Our study shows that the neonatal outcomes and risk of congenital malformations were similar between the PPOS and conventional GnRH-a short protocol. However, multiple pregnancy led to a higher likelihood of birth defects.

## Abstract 2

Supplemental Digital Content is available in the text

## 1 Introduction

Assisted reproductive technology (ART), such as in vitro fertilization (IVF) and intracytoplasmic sperm injection (ICSI), has been increasingly used to assist infertile couples in modern obstetrics. Since the birth of the first IVF-conceived infant in the United Kingdom in 1978, the number of pregnancies and births due to the use of this technology has risen exponentially.^[1,2]^ To date, this technology has resulted in more than 5 million infants born globally.^[3]^

Several studies have focused on ART-related side effects in women and the potential impacts of ART on their offspring. Although IVF complication rates are low, concern about the risks of some nonphysiological treatment procedures and the effects on the internal environment, such as ovarian stimulation regimens, high estrogen levels, and ovarian hyperstimulation syndrome (OHSS), continues to increase.^[4,5]^ Great efforts and innovations, such as the use of gonadotropin-releasing hormone-agonist (GnRH-a) instead of human chorionic gonadotropin (hCG) as the ovulatory trigger and the adoption of a freeze-all strategy, have been made to optimize the ovarian stimulation regimens and treatment strategies.^[6,7]^

In 2015, we reported a new regimen for controlled ovarian stimulation (COS), namely progestin-primed ovarian stimulation (PPOS), and oral progestin was first introduced into the COS procedure to prevent a premature LH surge in this novel regimen.^[8,9]^ Additionally, an internal LH surge could be induced by using GnRH-a to trigger ovulation. Together with the freeze-all strategy, 150 women who received PPOS had a satisfactory clinical pregnancy rate of 47.8%, and an implantation rate of 31.9% was obtained at that time.^[8]^ Compared with conventional COS regimens, the application of PPOS has the advantages of an oral administration route, more control over LH levels, and a lower risk of OHSS.^[10,11]^

The health of a child born following ART using any new procedure or compound is paramount. Any ovarian stimulation protocol for IVF/ICSI should not compromise the health of the mother during pregnancy or that of the infant born to the mother.^[12,13]^ Thus, a concern that accompanies the desire to incorporate this new regimen into routine practice regards whether the PPOS regimen is safe to the baby. In our previous preliminary study, more than 200 infants born from the PPOS regimen were followed up, and similar neonatal outcomes between the PPOS protocol and the traditional protocol were found.^[14]^ However, the relative small sample size and lack of an analysis of risk factors on congenital malformations limited the findings in our previous study. To date, this unique PPOS regimen has resulted in more than 800 infants born after embryo vitrification. Therefore, a larger cohort study is needed to systematically assess the safety of this new ovarian stimulation regimen.

Routine IVF procedures—using GnRH agonists or antagonists for the prevention of a premature LH surge—have become accepted as safe. This acceptance was based on the initial reassuring results of most follow-up studies, either prospective or retrospective.^[15,16]^ In consideration of their safety statistics for offspring, we selected the standard GnRH-a short protocol as the control group to evaluate the safety for offspring born from IVF-ET after PPOS.

## 2 Materials and methods

### 2.1 Study design and participants

This retrospective cohort study was conducted in the Department of Assisted Reproduction of the Ninth People's Hospital of Shanghai Jiao Tong University, School of Medicine. The study was approved by the ethical review committee of the Ninth People's Hospital of Shanghai and the Shanghai Jiaotong University Medical Centre, China.

Infertile couples, who underwent IVF or ICSI treatment with frozen-thawed embryo transfer (FET) using PPOS or the standard GnRH-a short protocol, were recruited for the study. These patients underwent the procedures from January 1, 2014 to July 1, 2016, leading to births between August 1, 2014 and April 1, 2017. Mothers with reported maternal diseases, such as gestational diabetes mellitus, hypertension, thyroid disorders, intrahepatic cholestasis of pregnancy or adverse environmental exposure during pregnancy, were excluded from this study because of the possible association of these factors with birth defects.^[17–20]^ The final data, involving 1589 live-born infants, were stratified into 2 groups according to the method of ovarian stimulation: 855 births after PPOS and 734 births after the short protocol. The study design and participant selection procedure are presented in Supplemental Figure 1.

### 2.2 Regimens

The details of the ovarian stimulation regimen for PPOS have been described in our previous publications.^[9]^ Briefly, ovarian stimulation was initiated for patients via the intramuscular injection of 150 or 225 IU of human menopausal gonadotropin (hMG; Anhui Fengyuan Pharmaceutical Co. Ltd, Hefei, China) and the simultaneous oral administration of 100 mg Utrogestan (Laboratories Besins International, Paris, France) daily, starting from day 2 or 3 of the menstrual cycle until the trigger day. The doses of hMG were adjusted after 5 to 7 days according to the patient's ovarian response.

In the short protocol, ovarian stimulation was carried out using a daily dose of 0.1 mg of triptorelin (Decapeptyl, Ferring Pharmaceuticals, Germany), injected subcutaneously, starting on day 2 or 3 of the natural cycle and continuing until the trigger day. This treatment was accompanied by the intramuscular injection of 150 IU or 225 IU of hMG, beginning on the same day as the first triptorelin administration. The doses of hMG were adjusted after 5 to 7 days according to the patient's ovarian response.

Monitoring was performed using transvaginal ultrasound scanning of the ovaries and serum estradiol measurements. When one dominant follicle reached 20 mm or when at least 3 follicles reached diameters of 18 mm, final oocyte maturation was induced with 0.1 mg of triptorelin for PPOS or 5000 IU of hCG (Lizhu Pharmaceutical Trading Co., Zhuhai, China) for the short protocol. Transvaginal ultrasound-guided oocyte retrieval was conducted 34–36 hours later after maturation induction. All follicles with diameters of more than 10 mm were retrieved. Flushing Medium (Origio Medical Company, Denmark) was used for oocyte retrieval, and Human Tubal Fluid (HTF; Irvine Scientific, CA) with 10% Serum Substitute Supplements (SSS; Irvine Scientific, CA) was used as the oocyte collection and insemination medium. Fertilization was carried out in vitro by either IVF or ICSI, depending on the semen parameters. The embryos were cultured in 10%SSS-supplemented Continuous Single Culture medium (CSC; Irvine Scientific, CA).

The day-3 embryos from each IVF/ICSI treatment cycle were examined according to Cummins’ criteria.^[21]^ Grade I and II embryos, which were regarded as top-quality, were frozen by vitrification. Grades III and IV embryos were placed in extended culture until the blastocyst stage. During this stage, only morphologically good blastocysts were frozen. The freezing and thawing procedure for embryos, embryo and endometrial synchronization procedure, and timing of ET are described elsewhere.^[22]^ In all FET cycles, no more than 2 thawed embryos were transferred according to the patient's intention. Mixed ET cycles from different ovarian stimulation protocols were excluded from the study. Once pregnancy was achieved, progesterone supplementation was continued until 10 weeks of gestation.

### 2.3 Outcome assessment

The couples completed a telephone interview during each stage of pregnancy up to 1 month after delivery. As previously reported, the interview questionnaire included the following information: a wide range of preconception and pregnancy exposures, pregnancy complications, gestational week, mode of delivery, birth date and locality, birth weight and length, infant sex, congenital malformations, and neonatal diseases, if any. For neonates born in hospital, a detailed physical examination was performed at birth and written reports were obtained from the pediatrician. For live-born infants with birth defects, case information was collected by a specially designated nurse to ensure that the infants met the case definition of the Chinese Birth Defects Monitoring Program.^[23]^

The outcomes were defined based on the International Committee for Monitoring Assisted Reproductive Technology and the World Health Organization revised glossary of ART terminology 2009.^[24]^ “Conception” was defined as a positive serum level of hCG. “Biochemical pregnancy” was defined as any miscarriage without any evidence of a fetal sac on transvaginal ultrasonography, but with a positive serum hCG pregnancy test. “Clinical pregnancy” was defined as the detection of a gestational sac via transvaginal ultrasonography. “Live birth” was defined as the complete expulsion or extraction of the fetus from its mother, followed by breaths or other evidence of life, such as a heartbeat, umbilical cord pulsation, or definite movements of voluntary muscles. “Ongoing pregnancy” was defined as an intrauterine pregnancy with fetal heart motion, with the absence of labor by the end of our research period. “Early neonatal death” was defined as the death of a live-born baby within 7 days of birth. “Congenital anomalies” were defined as all structural, functional, and genetic anomalies diagnosed in aborted fetuses, at birth or during the neonatal period. Congenital malformations were classified according to the International Classification of Diseases Q codes (Q00–Q99), tenth edition.^[25]^

### 2.4 Statistical analysis

Statistical analysis was performed using SPSS version 17.0 (SPSS Inc., Chicago, IL). The normality of continuous variables was tested using the Shapiro–Wilk test. Continuous variables were compared via Student's *t*-tests if the normality assumption was true; otherwise, the Kruskal–Wallis test was applied. Proportions were compared using Fisher's exact test or the chi-square test when appropriate.

A random effects logistic regression model was performed to quantify the effects of related factors on the congenital malformations. The potential factors in the model included maternal age, BMI, infertility duration, method of insemination (IVF*/*ICSI), singleton*/*multiple pregnancy, infant sex (male*/*female), type of transferred embryo (cleavage-stage embryo*/*blastocyst), number of transferred embryos per cycle and ovarian stimulation method (PPOS*/*short protocol). The effects of risk factors on congenital malformations were expressed through an adjusted odds ratio (OR) and 95% confidence interval (CI). Differences were considered statistically significant when the *P*-value was <.05.

## 3 Results

A total of 2076 conception cycles were preliminarily selected from our database, including 1107 cycles from the PPOS protocol and 969 cycles from the GnRH-a short protocol. According to the previously described access standards, 1657 clinical pregnancy cycles were ultimately enrolled (872 cycles from the PPOS and 785 cycles from the short protocol). The 1657 clinical pregnancies consisted of 1333 live births and ongoing pregnancies, 265 pregnancy losses, 32 ectopic pregnancies, 1 stillbirth, and 28 cases that were lost to follow-up, resulted in a total of 1589 live-born infants (1258 live-birth cycles). Among them, 659 pregnancies led to the births of 855 live-born neonates after treatment with the PPOS protocol; 599 pregnancies led to the births of 734 live-born infants after treatment with the GnRH-a short protocol. The evolution of the pregnancies followed, and the numbers of live-born and stillborn infants are presented in Table 1 and Supplemental Figure 1. As shown in Table 1, no obvious differences were evident between the PPOS group and the short protocol group regarding the rates of biochemical pregnancy, pregnancy loss, ectopic pregnancy, still birth, live birth and ongoing pregnancy, or loss to follow-up. These results indicated that comparable percentages of pregnancies led to the live births of infants from the 2 regimen groups.

**Table 1:**
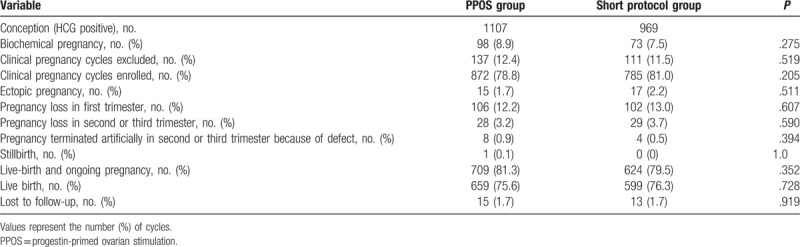
Number and evolution of pregnancies in the PPOS group and short-term protocol group.

Table 2 shows the maternal and cyclic characteristics of the live-birth cycles for both groups. The mean maternal age in the PPOS group was notably lower than that in the short-term group (32.9 ± 3.9 and 34.9 ± 3.7, respectively, *P < *.001). Other maternal characteristics, such as BMI, infertility duration, and the proportion of nulliparity were not significantly different between the 2 groups (21.3 ± 2.9 vs 21.4 ± 2.8, 3.3 ± 2.3 vs 3.7 ± 3.0, 90.9% vs 89.3%, respectively, *P* > 0.05). The cause of infertility and the origin of spermatozoa were also comparable between the 2 groups (*P*>0.05). IVF was applied most frequently in both groups (72.9% for PPOS group, 72.0% for short protocol group, *P* = 0.630), whereas the rate of blastocyst transfer in the PPOS group was significantly lower than that in the short protocol group (7.9% vs 14.8%, *P < *.001).

**Table 2:**
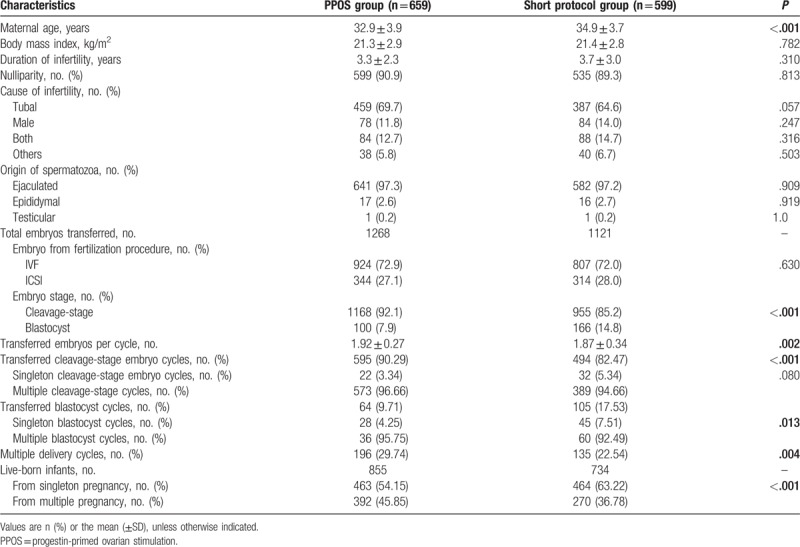
Maternal and cyclic characteristics of the live-birth cycles.

The details of the neonatal outcomes regarding gestational age, birth weight, birth length, and sex are summarized in Table 3. Data are provided separately for singleton and multiple births. As the results show, no notable differences in these characteristics were evident between the PPOS group and short protocol group. The overall incidence of neonatal death in live-born infants was also similar between the 2 groups (*P* > .05). These results indicated that comparable neonatal outcomes were achieved for both groups.

**Table 3:**
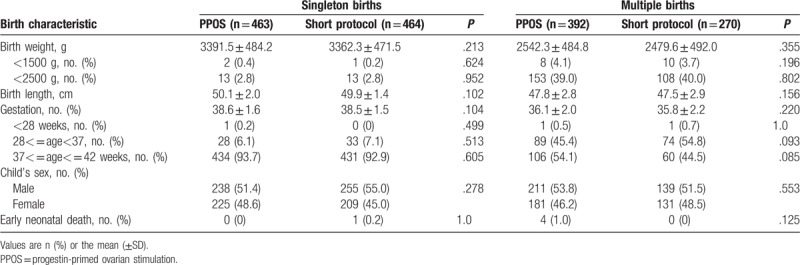
Characteristics of the births in the PPOS protocol group and GnRH-a short protocol group.

According to the definition in the International Classification of Diseases, a total of 25 cases (1.57%) among all live-born infants qualified as having congenital defects. As shown in Table 4, defects were observed in 13 out of 855 infants (1.52%) in the PPOS group and in 12 out of 734 infants (1.63%) in the short protocol group, and the difference was not statistically significant (*P* = .855). Comparisons between the groups regarding birth defects according to singletons, multiples, and neonatal sex were carried out, and the analysis showed no significant difference. Detailed information regarding the detected malformations according to the various organ systems is presented in Table 4. In both groups, the congenital defects were most frequently cardiac and vascular congenital disorders (0.94% for the PPOS group and 0.54% for the short protocol group); the second most frequent defects were gastrointestinal tract congenital disorders (0.41% as major for the short-term group) and musculoskeletal system congenital disorders (0.35% as major for the PPOS group). No significant difference was found between the types of malformations (e.g., circulatory system, digestive system, and chromosomal abnormalities) for both groups.

**Table 4:**
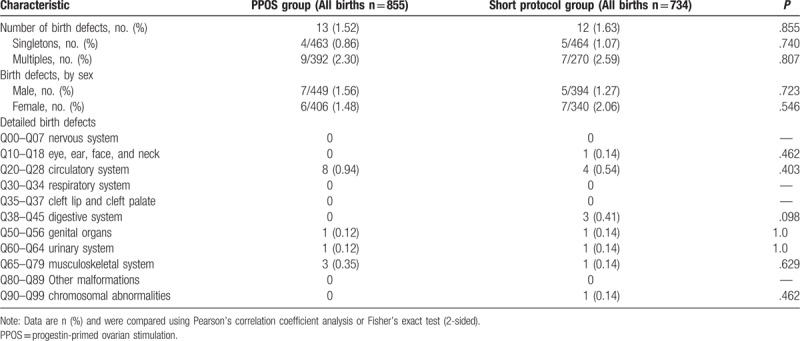
Incidence of birth defects in live-born infants and types of malformations according to the classification from code Q00–Q99 in the International Classification of Diseases, tenth edition.

Table 5 presents the results of a multivariate logistic regression model for the factors that may have influenced congenital malformations in live-born infants. As the results show, no associations were found between congenital malformations and maternal age, BMI, the duration of infertility, insemination method, infant sex, embryo stage at transfer, the number of embryos transferred per cycle or ovarian stimulation regimen. However, a statistically significant increase in the probability of an adverse outcome for multiple births was observed. Compared with singletons, multiples were 3.14 times more likely to experience an adverse outcome (OR: 3.14, 95% CI: 1.25–7.88; *P* = .015).

**Table 5:**
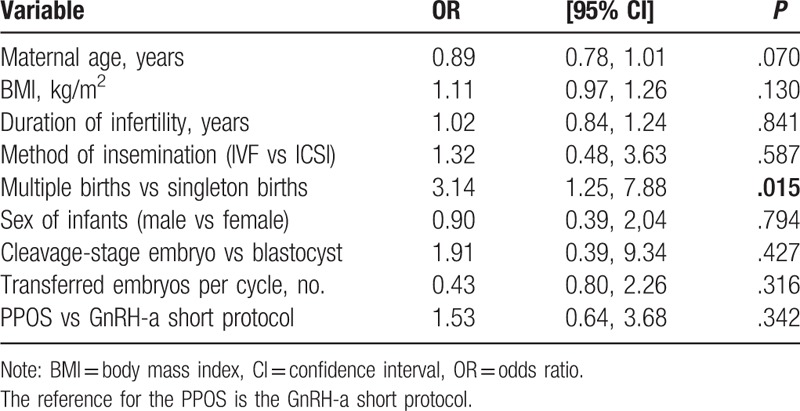
Random effects logistic regression of congenital malformation in live-born infants.

## 4 Discussion

In this study, the infant follow-up data from the large retrospective cohort study showed that the health of the 855 live-born infants conceived after treatment with the novel PPOS protocol was no different from that of the 734 live-born infants conceived after COS with the conventional GnRH-a short protocol. There were no treatment-related differences in neonatal characteristics or in the incidence of birth malformations. The rates for congenital malformations were not significantly different between the 2 groups, and the overall rate of congenital malformations of 1.57% observed here concurred with the rate of 1.11%-1.58% reported in a Chinese population-based study for birth defects occurring in infants, conceived by IVF treatment, within 7 days after delivery.^[26]^

Progesterone soft capsules (Utrogestan), containing natural micronized progestin, are usually used to support luteal function during pregnancy. Studies have shown that Utrogestan use is not associated with an increased risk of congenital malformation.^[27,28]^ In the PPOS regimen, progestin was first used in COS during IVF treatment in combination with the freeze-all strategy. Our previous studies have shown that progestin was an effective oral alternative for the prevention of a premature LH surge and could achieve comparable pregnancy outcomes to those of the short protocol in infertile woman. Nevertheless, concerns about the unconfirmed effects of PPOS on oocyte and/or embryo quality when the progestin level is high during COS still exist.^[29]^ Available evidence on this point is sometimes varied and even contradictory.

During folliculogenesis, the oocyte gains developmental competence and subsequently becomes a fully mature oocyte with the capacity to be fertilized and develop into a high-quality embryo.^[30]^ Any procedures or compounds that disturb the oocyte maturation process may damage its developmental competence. Zavareh and Salehnia^[31]^ reported that the addition of progestin to the in vitro maturation media of mouse GV oocytes significantly decreased the maturation rate and that the GV arrest rate increased. Fukui et al^[32]^ also demonstrated that progestin supplementation of IVM culture systems decreased the rate of bovine oocyte maturation. By contrast, Carter et al^[33]^ showed that the addition of progestin to the culture medium did not affect the proportion of in vitro matured/in vitro fertilized zygotes that developed to the blastocyst stage in vitro. In rhesus monkeys, an improvement in in-vitro oocyte development was demonstrated in the presence of progestin and estrogen.^[34]^ Overall, the literatures do not provide clear evidence on this point, and these inconsistent data may be due to differences in the experimental strategies that used.

In 2014, we first demonstrated that ovarian stimulation could be started routinely in the luteal phase using letrozole and exogenous gonadotropins, and the regimen was named luteal-phase ovarian stimulation (LPS).^[22]^ A follow-up study of more than 500 infants born from LPS showed that the differences in birth defects observed between LPS and conventional ovarian stimulations were not significant, demonstrating that a naturally high progestin status in the luteal phase did not increase the risk of congenital malformations.^[23,35]^ Therefore, data from the LPS provided evidence regarding the safety of progestin for human oocyte development. Even so, compared with the physiological progestin secreted by the corpus luteum during LPS, the effect of exogenous progestin, administered by daily oral administration of 100 mg Utrogestan, which can achieve the serum level of progestin during the luteal phase,^[36]^ on the safety of oocyte development still needs to be examined further. Therefore, we followed up on the neonatal outcomes and live-birth defects of infants born from PPOS, as data on the occurrence of malformations in offspring are the most important standards for assessing potential adverse effects of a new COS regimen.

In this large retrospective cohort study, we selected the classic GnRH-a short protocol as a control to investigate the safety of PPOS treatment for IVF or ICSI. The use of a short protocol has been proposed as a COS regimen in the subgroup of women with advanced age and/or decreased ovarian function.^[37]^ This selection approach may explain why women in the short protocol group were older (34.9 ± 3.7 years) than those in the PPOS group (32.9 ± 3.9 years, *P < *.001) and may explain the higher proportion of single blastocyst transfer cycles in the short protocol group than in the PPOS group (7.51 and 4.25%, *P < *.001). The follow-up data of the 855 live-born infants conceived after treatment with PPOS and the 734 live-born infants conceived after the short protocol showed that the live-birth rates, gestations, birth weights and lengths, infant sexes, congenital malformations, and stillbirths or neonatal deaths were similar between both groups. These findings indicated that there were no obvious differences in the neonatal characteristics or in the incidence of birth defects between the novel PPOS regimen and the classic ovarian stimulation regimen.

The prevalence estimates of birth defects were further adjusted for 9 covariates, as shown in Table 5. Unsurprisingly, multiple pregnancies were found to be a significant risk factor for congenital malformation. Multiple births have not only a higher risk of preterm delivery, low birth weight, and neonatal mortality, but also a higher risk of birth defects, such as cardiovascular defects, central nervous defects, and alimentary tract defects, than singletons.^[38,39]^ The significantly increased risk of birth defects with multiple births might be explained in several ways. First, multiple pregnancy significantly increases the risk of preterm delivery. Therefore, malformations associated with prematurity, such as patent ductus arteriosus and exomphalos, are increased in twins, and at least a part of the increase is due to prematurity. The second is that the crowded intrauterine space may cause positional defects so that multiple births are more likely to have mechanically induced defects. Finally, mothers with multiple fetuses may lack sufficient nutritional supply, adversely affecting normal fetal development. Therefore, multiple births is unequivocally a risk factor for birth defects. This finding may lead couples to favor elective, single ET, and couples undergoing ART should be made aware of the known increased risk of birth defects associated with a twin birth.

Some meta-analyses have suggested that infants, both singletons and multiples, born following ART are at increased risk of perinatal complications and birth defects compared with spontaneous conceptions.^[39,40]^ Meanwhile, the latest available review on the outcome of assisted reproduction concludes that some of the risks to infants born following ART do not arise as a result of the applied techniques or treatments but rather from the underlying health risks of being subfertile.^[41,42]^ Therefore, subfertile couples should improve their health statuses before undergoing ART.

In this study, all infants were born from FET cycles, which have been reported to have better obstetric and perinatal outcomes, compared with fresh ET cycles.^[43]^ Embryos that survive the freeze-thaw process are less likely to be aneuploidal, and therefore are of superior quality. Alternatively, or in addition, developing embryos in FET cycles may be shielded from the excessive ovarian hormonal exposures, which could have various adverse effects on early embryo development.^[44]^

As this study is limited to neonatal information extracted from parent questionnaires rather than from direct access to medical records, minor birth defects might have escaped detection, although these issues are unlikely to have altered the infant birth characteristics. Additionally, no association was found between the maternl age and birth defects in the multivariate logistic regression model in this study, however, previous studies have shown that advanced maternal age was associated with higher risks of birth defects. Although the effects of the majority of confounders were controlled, a potential bias cannot be excluded in this retrospective study. Therefore, a more rigorous prospective randomized controlled evaluation trial is necessary in the near future.

In conclusion, our available data do not indicate an elevated rate of birth abnormalities after PPOS compared with the classic COS regimen. In addition, long-term follow-up for the health of children born after PPOS is needed to further support the safety of this new treatment option in IVF.

## Acknowledgments

We gratefully acknowledge all the staff of the department of assisted reproduction in Shanghai Ninth People's Hospital for their support and cooperation.

## Author contributions

**Conceptualization:** Yonglun Fu, Yanping Kuang.

**Data curation:** Ningling Wang, Jiaying Lin, Yong Fan.

**Methodology:** Qianqian Zhu.

**Project administration:** Yun Wang, Yonglun Fu, Yanping Kuang.

**Writing – original draft:** Ningling Wang.

**Writing – review & editing:** Ningling Wang, Yonglun Fu, Yanping Kuang.

## Supplementary Material

Supplemental Digital Content
