# Supplementary material for: Comparison of neonatal outcomes and live-birth defects after progestin-primed ovarian stimulation versus conventional ovarian stimulation for in vitro fertilization: A large retrospective cohort study
Source: Medicine (Baltimore). 2018 Aug 24;97(34):e11906. doi: 10.1097/MD.0000000000011906 (PMC6112954; doi:10.1097/MD.0000000000011906)
Supplement: Supplemental Digital Content [file medi-97-e11906-s001.doc]

Supplemental Figure 1: Flow chart of the study.


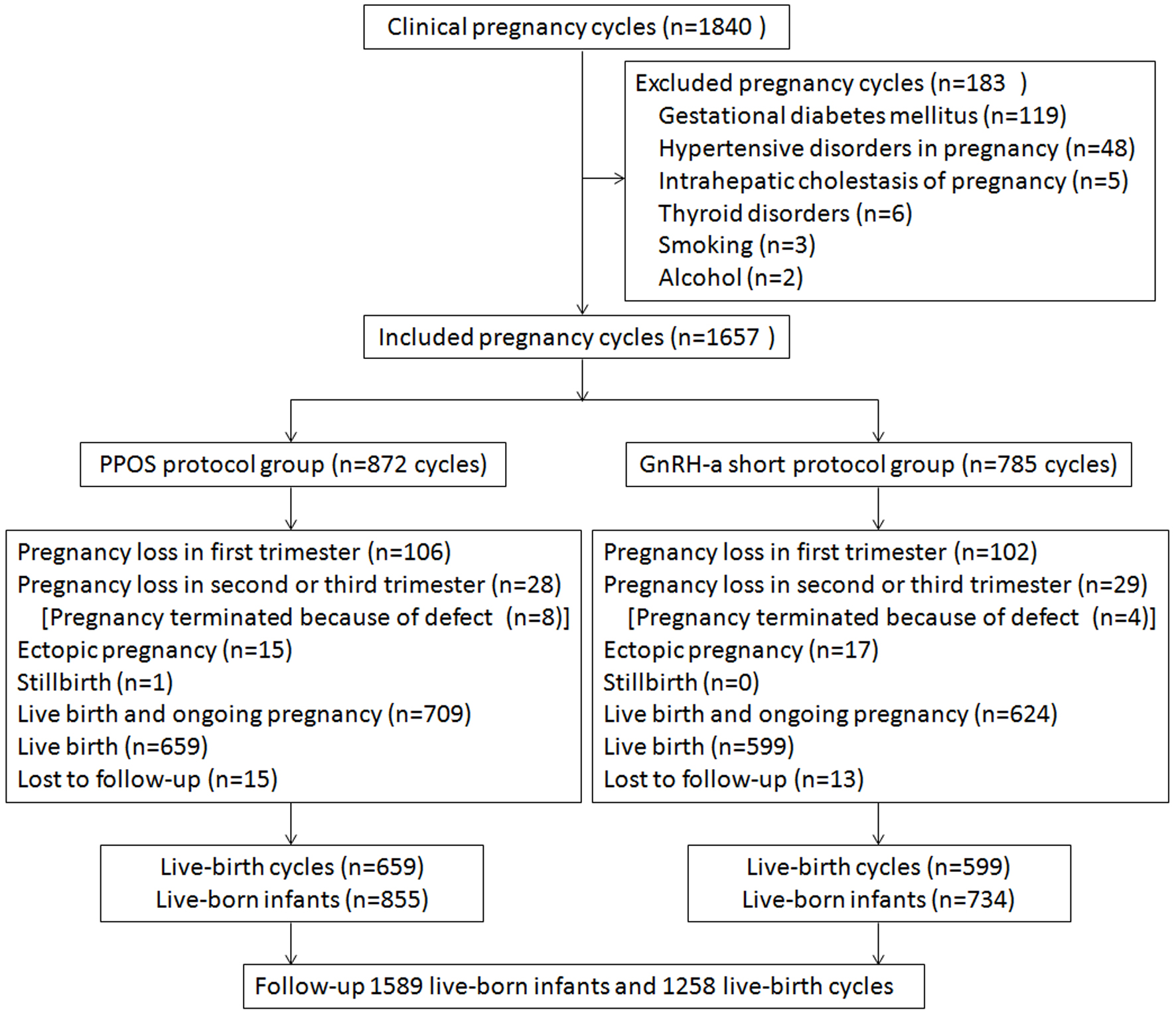


No obvious difference was found between the PPOS group and the short protocol group regarding the rates of pregnancy loss in the first trimester, pregnancy loss in the second or third trimester, ectopic pregnancy, stillbirth, live birth and ongoing pregnancy and loss to follow-up (P>0.05). For pregnancy loss in the second or third trimester, among the two groups, there were a total of 12 medical treatment-induced terminations of pregnancy because of fetal malformations: 8 in the PPOS group and 4 in the short protocol group. The difference was not significant (P>0.05). The malformations were classified as congenital heart disease (2 cases), chromosomal abnormalities (4 cases), nervous system disease (1 case), musculoskeletal system disease (1 case), hydrops fetalis (1 case), hygroma (1 case), cleft lip and cleft palate (1 case) and another malformation.
